# Fast Thermoelectric Responses from Unconventional Na‐I Stoichiometry in Reduced Graphene Oxide Films

**DOI:** 10.1002/advs.202515896

**Published:** 2025-12-05

**Authors:** Xinming Xia, Wenjin Luo, Tao Wang, Yunzheng Zhang, Jie Jiang, Pei Li, Liuhua Mu, Liang Chen, Yusong Tu

**Affiliations:** ^1^ School of Physical Science and Technology & Microelectronics Industry Research Institute Yangzhou University Yangzhou 225009 China; ^2^ School of Physical Science and Technology Ningbo University Ningbo 315211 China

**Keywords:** 2D materials, cation‐π interaction, iodide, thermoelectricity, unconventional stoichiometry

## Abstract

Thermoelectric materials composed of 2D structures have received extensive attention and research due to their potential properties. The development of novel 2D materials will provide many valuable technological characteristics for thermoelectric applications. Here, fast thermoelectric responses are reported from unconventional Na‐I stoichiometry in reduced graphene oxide films (Na‐I@rGO). The thermoelectric mechanism is based on the Seebeck effect caused by the heterogeneous structure between the rGO layers from Na_2_I and NaI. The formation of this heterogeneous structure is attributed to the gravity‐driven ion permeation during the preparation process and cation‐π interactions. Na‐I@rGO film exhibits a significant thermoelectric current at a temperature difference (Δ*T*) of 40 K (peak current ∼650 nA). The Seebeck coefficient of Na‐I@rGO film is ≈22.7 µV K^−1^ for a Δ*T* range of 2−40 K. Importantly, Na‐I@rGO film has a fast response (0.6 s) as a self‐powered sensing device, which is comparable to that of most other reported classical thermoelectric materials. The Na‐I@rGO sensor can be suitable for fast detecting transient extreme temperature variations, such as flame and liquid nitrogen. These findings can provide inspiration for the design of 2D thermoelectric structures on graphene films.

## Graphical abstract

By coating the graphene oxide suspension and the dilute NaI solution layer‐by‐layer from bottom up, this work achieves a heterogeneous structure from asymmetry of the up and bottom surface Na and I atomic ratios in reduced graphene oxide (rGO) films, which exhibits fast thermoelectric responses.

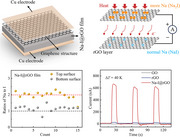

## 1 Introduction

Unconventional thermoelectric materials or concepts are capable of converting various types of particles that transmit charge from heat flux to electric current, which helps to expand the applications of envisioned thermoelectric materials far beyond the simple conversion of heat into electricity.^[^ ^1^, ^2^, ^3^, ^4^, ^5^, ^6^ ^]^ These materials have great promise for energy harvesting and sensing applications. However, despite continuous progress, finding novel and valuable thermoelectric materials remains a persistent challenge. Thermoelectric materials composed of 2D structures have received extensive attention and research due to their potential properties,^[^ ^7^, ^8^, ^9^, ^10^, ^11^, ^12^ ^]^ such as graphene, black phosphorus, transition metal dichalcogenides, group IVA‐VIA compounds, and MXenes. The development of novel 2D materials will provide many valuable technological characteristics for thermoelectric applications, such as micro‐nano dimensions,^[^ ^13^ ^]^ easy large‐scale fabrication,^[^ ^14^ ^]^ low cost, and abundant raw materials.^[^ ^15^ ^]^

Recently, unconventional stoichiometries of metal halide on the graphene, such as Na_2_Cl, Na_3_Cl,^[^ ^16^, ^17^ ^]^ Li_2_Cl^[^ ^18^ ^]^ and CaCl,^[^ ^19^ ^]^ have been explored at ambient conditions based on cation‐π interactions.^[^ ^20^, ^21^ ^]^ They exhibit unique electronic structures that confer them versatile electronic, magnetic, and optical properties. We note that the formation of unconventional Na‐I stoichiometry on graphene oxide (GO) is accompanied by a lift in its conductivity due to the reduction of GO by the iodide ion.^[^ ^22^ ^]^ In addition, previous studies have theoretically predicted the alkali metal halide layer structures, especially the iodide layers, with excellent thermoelectric properties.^[^ ^9^ ^]^ Accordingly, we believe that the unconventional Na‐I stoichiometry in reduced graphene oxide (rGO) layers has potential applications in the field of thermoelectricity and provides inspiration for the expansion of thermoelectric structures on graphene films.^[^ ^12^ ^]^

Here, we report fast thermoelectric responses from unconventional Na‐I stoichiometry in rGO films (Na‐I@rGO). The thermoelectric mechanism is based on the Seebeck effect caused by the heterogeneous structure between the rGO layers from Na_2_I and NaI. The formation of this heterogeneous structure is attributed to the gravity‐driven ion permeation during the preparation process and cation‐π interactions. Under the temperature difference (Δ*T*) of 40 K, Na‐I@rGO film exhibits a significant thermoelectric current (peak current ∼650 nA), while the current responses of GO and rGO are negligible. The Seebeck coefficient of Na‐I@rGO film is ≈22.7 µV K^−1^ for a Δ*T* range of 2−40 K. Importantly, Na‐I@rGO film has a fast response (0.6 s) as a self‐powered sensing device, which is comparable to that of most other reported classical thermoelectric materials. The Na‐I@rGO sensor can be suitable for fast detecting transient extreme temperature variations, such as flame and liquid nitrogen.

## 2 Results and Discussion

Freestanding films were prepared by a bottom‐up^[^ ^15^ ^]^ and layer‐by‐layer^[^ ^23^ ^]^ method for growing unconventional Na‐I stoichiometry in the rGO layers (Na‐I@rGO) from GO suspension and dilute NaI solution. The GO suspension was thoroughly cleaned and dialyzed to remove impurities. The 5 mg mL^−1^ GO suspension was uniformly coated on the polyimide (PI) substrate, dried at 60 °C to allow initial curing, and then coated with 0.05 m diluted NaI solution (200 µL) and dried at 60 °C. After three cycles, GO was coated on the product and dried at 60 °C. The Na‐I@rGO films were obtained after drying for 24 h. Next, the Na‐I@rGO films were analyzed by scanning electron microscopy‐energy dispersive X‐ray spectroscopy (SEM‐EDS), X‐ray diffraction (XRD), and X‐ray photoelectron spectroscopy (XPS).

**Figure 1:**
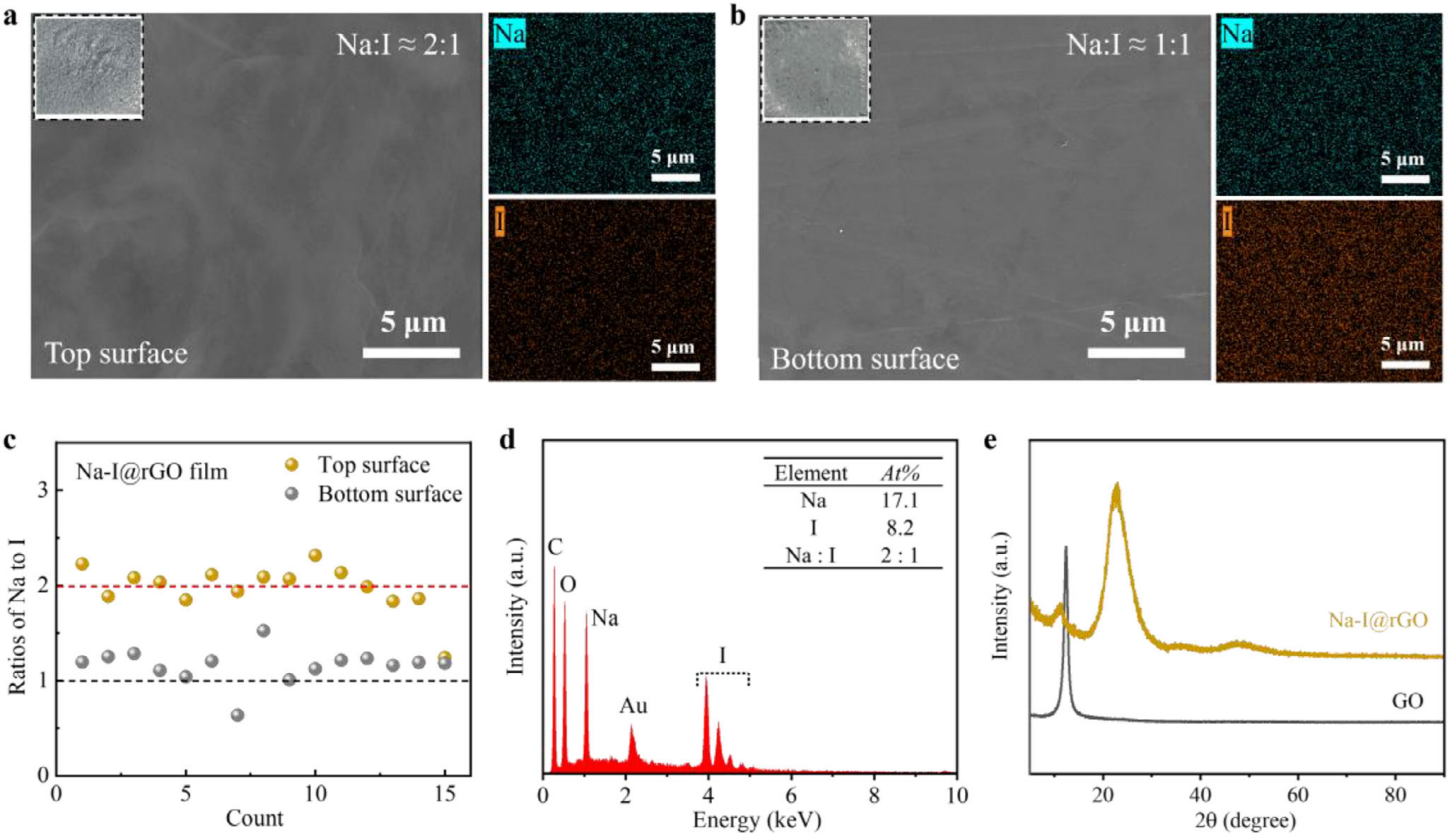
Unconventional Na‐I stoichiometry in rGO films. SEM images of the a) top and b) bottom surfaces of Na‐I@rGO film and corresponding EDS mappings. The insets are photographs of the film (1 cm × 1 cm). c) Atomic ratios of Na to I on the top and bottom surfaces of Na‐I@rGO film determined by SEM‐EDS analysis. Selected areas of EDS were randomly chosen from a large sampling region. d) Elemental spectral characteristic with a Na to I ratio of ≈2:1 in SEM‐EDS analysis (Au element is the coating material used for SEM sample preparation). e) XRD pattern of Na‐I@rGO film and GO film (the difference between the two films in the preparation process is only whether NaI solution is added).

The Na and I atomic ratios form a distinct asymmetry of the up and bottom surfaces in the Na‐I@rGO film. As shown in **Figure** **1**a,b, the EDS mapping results show that Na and I are evenly distributed in the graphene layers on the up and bottom surfaces of the film. Interestingly, the Na to I ratio is 2:1 on the up surface, whereas the ratio is 1:1 on the bottom surface. The physical appearances of the up and bottom surfaces of the Na‐I@rGO film are shown in the insets. Further measurement with a large number of random EDS selections demonstrated that Na: I was dominated by 2:1 on the up surface and 1:1 on the bottom surface over the test depth range of the µm (Figure 1c; Figure S1, Supporting Information). From the SEM‐EDS and XPS spectra of Na‐I@rGO film, we identified chemical species with no observable impurity (Figure 1d; Figure S2, Supporting Information). It should be noted that electron beams can cause damage to iodine, especially on the surface layer of NaI (at the nm depth), such as XPS. However, the detection results of SEM‐EDS are relatively accurate (at the µm depth) (Figure S2, Supporting Information).

The thickness of the Na‐I@rGO film is ≈10 µm and it has certain flexibility (Figure S3, Supporting Information). Since the Na‐I@rGO film was prepared by solution‐phase processing, the rGO layers from Na_2_I and NaI also form perfect ohmic contacts with near‐zero contact resistance. We observed that the ratio of Na to I in the cross‐section of the Na‐I@rGO film is 2:1 (Figure S4, Supporting Information). This indicates that the ratio of Na‐I (1:1) is concentrated on the bottom surface (within the detection µm depth range of SEM‐EDS). However, the asymmetric distribution of the rGO layers from Na_2_I and NaI in the film is significant and effective (Figure 1c).

The asymmetric Na‐I stoichiometry on the Na‐I@rGO film arises from gravity‐driven ion permeation, where the differential migration of Na and I ions through the GO film is governed by their specific ion‐π interactions. This affected the distribution of solution ions on the bottom surface as well as other positions (including the upper surface). Our previous density functional theory (DFT) calculations^[^ ^16^ ^]^ have revealed the specific cation and anion‐π interactions that govern this process, further demonstrating that these interactions differ between a single graphene layer and the interlayer space. This is consistent with the experimental results of the stoichiometry of Na‐I at the bottom surface (NaI) and other positions (Na_2_I) (Figure 1c; Figure S4, Supporting Information). In detail, in the process of Na‐I stoichiometry formation, due to charge transfer between the unoccupied valence orbital of Na^+^ and the delocalized π state of the aromatic ring structure in the graphene sheet (the cation‐π interaction between Na^+^/I^−^ and the graphene aromatic rings^[^ ^16^, ^21^ ^]^), unconventional structure or stoichiometry are formed, in which the ratios of the Na and I elements can be 1:1, 2:1, such as NaI and Na_2_I^[^ ^16^ ^]^.

Ultraviolet (UV) absorption spectroscopy provided further evidence for the presence of cation‐π interactions (Figure S5, Supporting Information). The UV spectrum of GO at ≈230 nm, which is assigned to a conjugate double bond of the aromatic group that easily generated π–π^*^ transition.^[^ ^24^ ^]^ Compared with the UV intensity of GO in H_2_O, the intensity of GO in Na^+^ solution markedly decreased, indicating that the conjugate double bonds of the aromatic group in GO are affected by the cation. The intensity of this interaction is directly related to the valence state and charge density of the cation.^[^ ^25^ ^]^ It should be noted that the UV of GO is enhanced at ≈230 nm due to I^−^. This is because I^−^ has a high polarizability, which enables it to donate its electron portion to the π^*^ antibonding orbital of GO, thereby enhancing the π–π^*^ transition.^[^ ^26^, ^27^ ^]^

In addition, iodide has a reducing effect. XRD results show that there is a significant difference in the film obtained with or without NaI solution during the preparation process (Figure 1e). Due to the instability of graphene iodide, it undergoes spontaneous decomposition, resulting in the rGO layers. The relevant mechanisms involved in the reduction process has been elucidated in detail in previous reports.^[^ ^22^ ^]^

**Figure 2:**
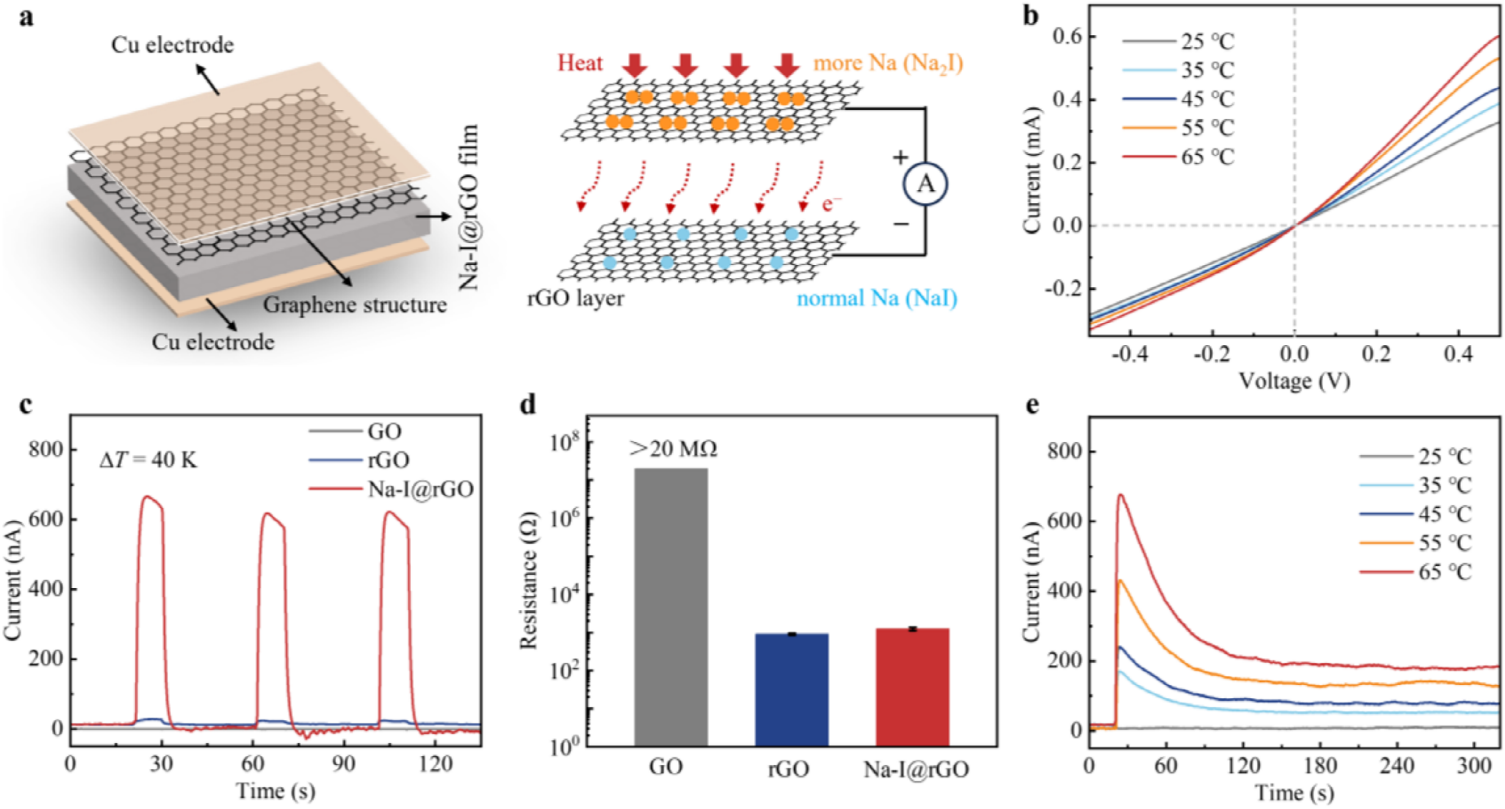
Thermoelectric effect of Na‐I@rGO films at room temperature. a) Schematic showing the Na‐I@rGO thermoelectric device structure and working mechanism. b) Voltage–current (*I–V*) characteristic curves (−0.5 to 0.5 V) during heating at different temperatures, compared to the room temperature of 25 °C. c) Thermoelectric response of the GO, rGO, and Na‐I@rGO films under intermittent heating (Δ*T* = 40 K, where 1 K = 1 °C). d) Resistance between the up and bottom surfaces of the GO, rGO, and Na‐I@rGO films determined by multimeter. Error bars indicate the standard deviation from five different samples. e) Thermoelectric response of the Na‐I@rGO film under constant heating compared to the room temperature of 25 °C.

Next, the Na‐I@rGO films were placed between conductive Cu/Cu electrodes as a thermoelectric device and were encapsulated by PI (**Figure** **2**a). The thermoelectric mechanism is based on the Seebeck effect caused by the heterogeneous structure between the rGO layers from Na_2_I and NaI. In this geometry, the direction of thermal and charge flow is perpendicular to the plane of the heterointerfaces, forcing carriers to sequentially traverse each interface. Unlike the parallel model of traditional planar heterojunctions, it is similar to the emerging series model of vertical heterojunctions.^[^ ^28^ ^]^ The observed Seebeck effect can be attributed to this interface‐dominated tandem accumulation mechanism. There are charge transfer differences of Na^+^‐π between the Na_2_I and NaI in the rGO layers. These charge differences lead to differences in their electronic properties^[^ ^16^, ^17^, ^19^ ^]^ because they have more Na (Na_2_I) than normal Na (NaI) in the rGO layers. Under the temperature difference (Δ*T*), thermal diffusion of electrons generates thermal voltage/current. The I‐V characteristics of the Na‐I@rGO film at different Δ*T* exhibit a rectification effect (Figure 2b), indicating that there is a certain degree of heterogeneity in the film.^[^ ^29^, ^30^ ^]^ The heterogeneity is believed to be caused by the Na‐I stoichiometric ratio on the up and bottom surfaces. As the Δ*T* increases, the slope of the I‐V curve shows varying degrees of increase, indicating enhanced thermoelectric coupling due to the rising Seebeck voltage in the Na‐I@rGO films.

Under the intermittent Δ*T* of 40 K, the thermoelectric responses of different graphene‐based films were observed. The film's devices were naturally placed on the heating plate, with the Δ*T* being the difference between the heating temperature and the air temperature. It was found that Na‐I@rGO film exhibited a significant thermoelectric current (peak current ∼650 nA), while the current responses of GO and rGO were negligible (Figure 2c). For pure NaI, its thermoelectric response was also almost negligible (Figure S6, Supporting Information). These films have dimensions of 1 cm × 1 cm × 10 µm. The room temperature resistance of Na‐I@rGO films is similar to that of rGO films (0.9–1.3 kΩ), both being much lower than that of GO films (>20 MΩ) (Figure 2d). It is noted that the energy harvesting performance of the Na‐I@rGO film is limited due to the moderate electrical conductivity (Figure S7, Supporting Information). Furthermore, Na‐I@rGO film can maintain a stable current output under continuous heating (Figure 2e), which confirms that its thermoelectric mechanism is due to electron transport rather than ion thermal diffusion. This is due to the accumulation of ions at the interface between the metal electrode and the electrolyte in ionic thermoelectricity, forming an electrical double layer, which only generates a transient current.^[^ ^1^, ^2^ ^]^

**Figure 3:**
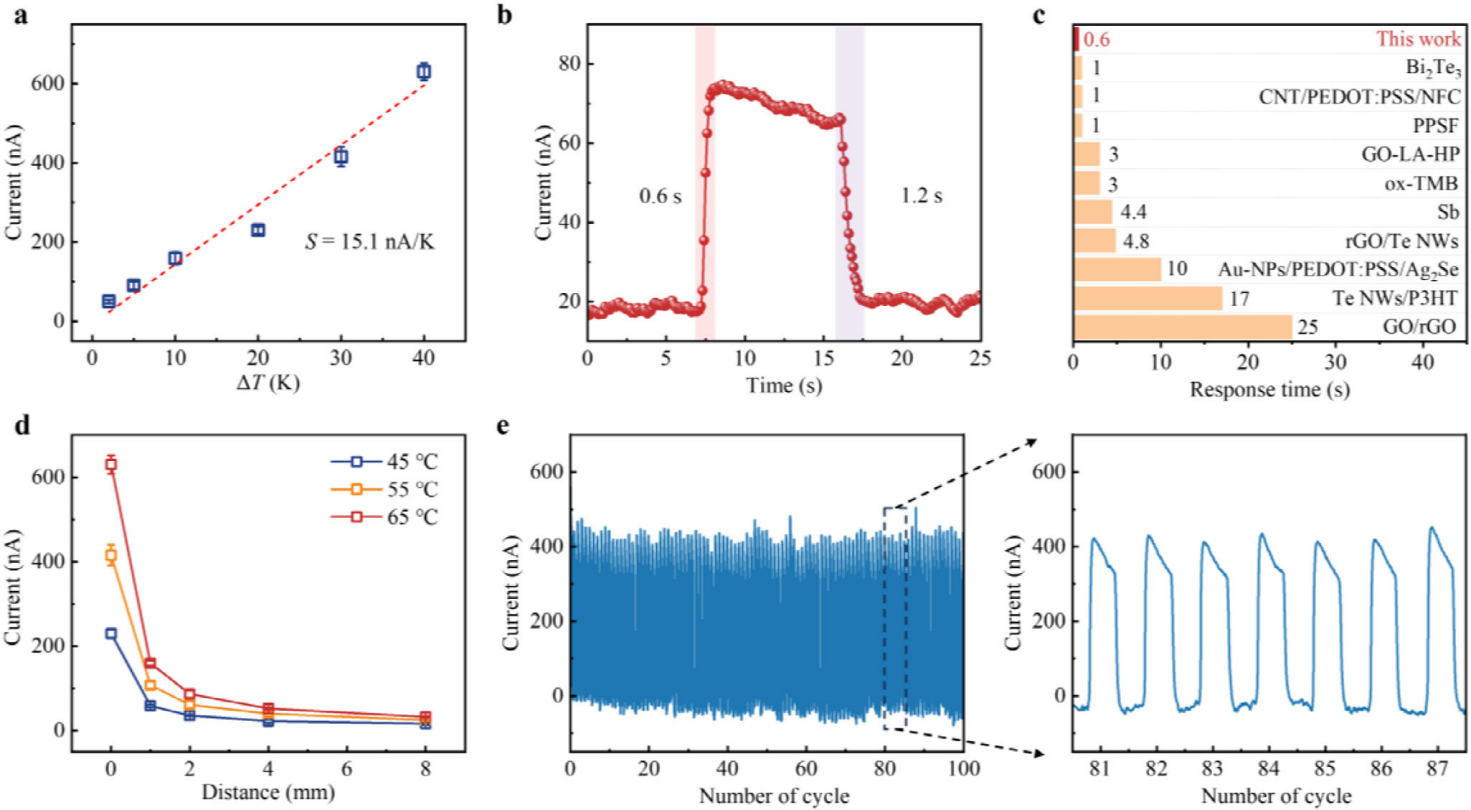
Thermoelectric sensing performance of Na‐I@rGO films at room temperature. a) Changes of response current under different Δ*T* (2, 5, 10, 20, 30, and 40 K, where 1 K = 1 °C) of the Na‐I@rGO sensor. The error bars indicate the standard deviation of three replicate measurements of the sample. b) Response and recovery times of Na‐I@rGO sensor by the rapid contact and separation of the sensor and heater (30 °C) manually. The current responses were measured with a time step of 0.1 s. c) Comparison of the response times of the Na‐I@rGO sensor with some reported classical thermoelectric materials, including Bi_2_Te_3_, carbon nanotube/poly(3,4‐ethylenedioxythiophene)poly(styrenesulfonate) /nanocellulose (CNT/PEDOT:PSS/NFC), PEDOT:PSS/spacer fabric (PPSF), GO‐ascorbic acid‐phenoxycycloposphazene (GO‐LA‐HP), oxidized 3,3′,5,5′‐tetrametylbenzidine (ox‐TMB), Sb, rGO/Te nanowires (rGO/Te NWs), gold nanoparticles/PEDOT:PSS/Ag_2_Se (Au‐NPs/PEDOT:PSS/Ag_2_Se), Te NWs /poly(3‐hexyl thiophene) (Te NWs/P3HT), and GO/rGO. d) Response current of the Na‐I@rGO sensor at different distances (*D* = 1, 2, 4, and 8 mm) with the 45, 55, and 65 °C heaters. The error bars indicate the standard deviation of three replicate measurements of the sample. e) Working stability tested over 100 contact‐separation cycles with a 55 °C heater, including an enlarged view of the corresponding area.

The Na‐I@rGO films can serve as a self‐powered sensing device^[^ ^5^ ^]^ and exhibit excellent thermoelectric sensing performance^[^ ^31^, ^32^ ^]^ (**Figure** **3**). Changes in the response current of the film under different Δ*T* provided by a heater at room temperature were determined by a current source meter (Figure S8, Supporting Information). Further, the sensitivity of a thermoelectric sensor is defined as *S* = *d*Δ*I*/*d*Δ*T* with a unit of nA/K, where Δ*I* is the relative change of the current, and *ΔT* is the applied temperature difference. As shown in Figure 3a, the sensitivity of the Na‐I@rGO sensor is 15.1 nA K^−1^ for a Δ*T* range of 2−40 K, and the Seebeck coefficient is ≈22.7 µV K^−1^ by ohmic transformation. It is noted that the Δ*T* is assumed to be the difference between the heater temperature and the air temperature. This, in general, would cause overestimation of temperature differences as real Δ*T* is smaller than that value. This means the actual Seebeck coefficient could be higher. The thicknesses of the Na‐I@rGO films have an influence on the thermoelectric sensing performance (Figure S9, Supporting Information). The thickness of the film can be controlled by gradually adding or reducing the NaI/GO solution layers. Increasing the thickness of the film will increase the bulk resistance of the film. Conversely, reducing the thickness of the film may have adverse effects on the formation of the Na_2_I/NaI interface and the reduction of GO.

Importantly, the sensor exhibits a fast response (0.6 s) and recovery (1.2 s) by rapidly contacting and separating a heater of ≈30 °C (Figure 3b). The response time is comparable to that of most other reported classical thermoelectric materials^[^ ^5^, ^31^, ^32^, ^33^, ^34^, ^35^, ^36^, ^37^, ^38^, ^39^ ^]^ (Figure 3c). The distance from the heater to the sensor affects the response current (Figure 3d). Longer distances reduced current by attenuating heat convection, while higher temperatures increased it by enhancing convection.^[^ ^40^ ^]^ In addition, the current response of the sensor remains highly stable and repeatable, exhibiting almost negligible drift when subjected to over 100 contact‐separation cycles from a 55 °C heater (Figure 3e).

**Figure 4:**
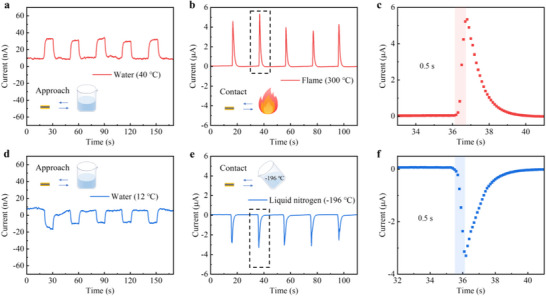
Thermoelectric sensing application of Na‐I@rGO films at room temperature. a) Response to current changes when a beaker with warm water approaches and leaves the sensor. b,c) Response current changes during transient flame contact and leave, and the corresponding amplification region shows its response time, with a time step of 0.1 s. d) Response to current changes when a beaker with cold water approaches and leaves the sensor. e,f) Response current changes during transient liquid nitrogen contact and leave, and the corresponding amplification region shows its response time, with a time step of 0.1 s.

The Na‐I@rGO sensors can be applied to detect temperature changes (**Figure** **4**). The periodic approach and leave of beakers containing warm or cold water for the sensor can be identified by detecting the changes in current response. Note that when the Δ*T* is reversed, the current is reversed at the same time, which is due to the fact that electrons always flow from the hot end to the cold end. In addition, the Na‐I@rGO sensor is suitable for detecting transient extreme temperature variations. When sudden flame (≈300 °C) or liquid nitrogen (−196 °C) comes into rapid contact with the sensor, the current signal responds rapidly, up to 5 or −3 µA, and the response time is only ≈0.5 s.

## 3 Conclusion

In summary, we have presented fast thermoelectric responses from unconventional Na‐I stoichiometry in rGO films. The thermoelectric mechanism is based on the Seebeck effect caused by the heterogeneous structure between the rGO layers from Na_2_I and NaI. The Na‐I@rGO film exhibits a significant thermoelectric current, and the Seebeck coefficient is ≈22.7 µV K^−1^ for a Δ*T* range of 2−40 K. Importantly, the Na‐I@rGO films exhibit excellent thermoelectric sensing performance in terms of self‐power, sensitivity, and response time. The Na‐I@rGO sensor can be suitable for fast detecting transient extreme temperature variations, such as flame and liquid nitrogen. These findings can provide inspiration for the design of 2D thermoelectric structures on graphene films.

## 4 Experimental Section

### Preparation and Characterizations

The GO suspension was prepared from natural graphite powder using the modified Hummers method. The GO suspension was thoroughly cleaned and dialyzed to remove impurities. Freestanding Na‐I@rGO films were prepared by a bottom‐up and layer‐by‐layer method for growing unconventional Na‐I stoichiometry in the rGO layers from GO suspension and dilute NaI solution. (detailed in Results and Discussion). In addition, GO films and rGO films were prepared as the control groups. GO films were prepared by replacing the dilute NaI solution with water, and rGO films were obtained by heating the GO films at 180 °C for 1 h.

Next, the Na‐I@rGO films were placed between conductive Cu/Cu electrodes as a thermoelectric device. Under the condition of ensuring no short circuit occurs, the electrodes have an area that is close to that of the surface of the film. The Cu/Na‐I@rGO film/Cu structure is encapsulated with PI and fixed onto a thin glass slide. They can then be further tested for their electrical properties or used as sensor devices. The thickness of the Na‐I@rGO films can be controlled by gradually adding or reducing the NaI/GO solution layers. The one, three, five, and seven layers of NaI/GO solution were successively added onto the GO layer, thereby preparing Na‐I@rGO films with thicknesses of 5, 10, 15, and 20 µm, respectively.

SEM micrographs and EDS were determined at room temperature by a Hitachi SU70 thermal field emission scanning electron microscopy operating at 15.0 kV. EDS were performed on a number of selected areas (≈1 µm^2^) on the surface. Selected areas of EDS were randomly picked from a large sampling region. Repeat the above operation to obtain the statistical results of the atomic fraction. XRD patterns of the films were determined at room temperature by a Bruker D8 Advance X‐ray diffraction in the 2θ range of 5°−90°. XPS spectra were determined by a Thermo Scientific K‐Alpha X‐ray photoelectron spectroscopy. UV absorption spectra were determined by a Nano‐500 micro‐spectrophotometer.

### Thermoelectric Response Measurement

All the measurements were performed under ambient conditions and at room temperature (≈25 °C). Response currents were measured by a current source meter provided by the CHI760E electrochemical workstation. The heat source was provided by a constant‐temperature heating plate.

The film devices were naturally placed on the heating plate, with the Δ*T* being the difference between the heating temperature and the air temperature. I‐V characteristic curves (−0.5 to 0.5 V) and response currents during heating at different temperatures (25, 35, 45, 55, and 65 °C) were determined by maintaining one surface of the film in continuous contact with the heat source. Changes of response current under different Δ*T* conditions compared to room temperature were determined by intermittent (10 s) contact with the heat source. In order to ensure the Δ*T* between the two ends of the sensor, it is generally placed at the heat source for 10 s and then cooled at ambient temperature for 30 s. The experiment was repeated three times to calculate the error. In addition, the response current was tested at the distance of the sensor from the heat source (1, 2, 4, and 8 mm). Response time and work stability were determined by the rapid contact and separation of the sensor and heat source manually. The response currents were measured with a time step of 0.1 s. Working stability was tested over 100 cycles with a cycle of 20 s.

### Applications of Thermoelectric Sensing

All the measurements were performed under ambient conditions and at room temperature (≈25 °C). A beaker containing water was periodically placed near the sensor at a distance of 1 mm, and the current characteristics were determined using a current source meter. The temperature of warm (40 °C) or cold (12 °C) water in the beaker was determined by a glass‐stem thermometer. In the test of extreme temperature changes, the high temperature provided by the flame is the temperature resistance value of the encapsulant of the sensor (≈300 °C), and the low temperature provided by the liquid nitrogen is −196 °C. The extreme temperature source was placed in direct and rapid contact with the sensor, and the contact was repeated periodically. The current characteristics were determined with a current source meter.

## Conflict of Interest

The authors declare no conflict of interest.

## Author Contributions

Y.T. and L.C. conceived the ideas. Y.T., L.C., and X.X. designed the experiments and co‐wrote the manuscript. X.X. carried out the experiments, collected and analyzed the data. W.L., T.W., and Y.Z. assisted with data collection. All authors discussed the results and commented on the manuscript.

## Supporting information



Supporting Information

## Data Availability

The data that support the findings of this study are available from the corresponding author upon reasonable request.
