# Supplementary material for: Fast Thermoelectric Responses from Unconventional Na‐I Stoichiometry in Reduced Graphene Oxide Films
Source: Adv Sci (Weinh). 2025 Dec 5;13(11):e15896. doi: 10.1002/advs.202515896 (PMC12931231; doi:10.1002/advs.202515896)
Supplement: Supplementary file 1 — Supporting Information [file ADVS-13-e15896-s001.docx]

Supporting Information

**Fast thermoelectric responses from** **unconventional Na-I stoichiometry in reduced graphene oxide films**

Xinming Xia^1,2^, Wenjin Luo^1,2^, Tao Wang^2^, Yunzheng Zhang^2^, Jie Jiang^2^, Pei Li^2^, Liuhua Mu^2^, Liang Chen^2,^*, and Yusong Tu^1,^*

*1. School of Physical Science and Technology & Microelectronics Industry Research Institute, Yangzhou University, Yangzhou 225009, China.*

*2. School of Physical Science and Technology, Ningbo University, Ningbo 315211, China.*

* Corresponding author. E-mail: ystu@yzu.edu.cn (Y.T.); liangchen@zafu.edu.cn (L.C.)

**Supplementary Text**

Preparation of the GO suspension.

The graphene oxide (GO) suspension was prepared from natural graphite powder using the modified Hummers method. Graphite powders were put into concentrated H_2_SO_4_, K_2_S_2_O_8_, and P_2_O_5_ solution with continuous stirring for several hours. Then the mixture was diluted with deionized (DI) water, centrifuged, and washed with DI water. After dried, the pre-oxidized graphite was obtained. Pre-oxidized graphite was oxidized in concentrated H_2_SO_4_ and KMnO_4_ at 6 ℃, and diluted with DI water at 20 ℃. The product was further oxidized with H_2_O_2_ and centrifuged and washed using HCl aqueous solution and DI water. Then, the product was desalted for two weeks using a dialysis bag with a molecular weight cut-off of 8000-14000 g/mol. The pure GO suspension (5 mg/mL) was obtained for further use.


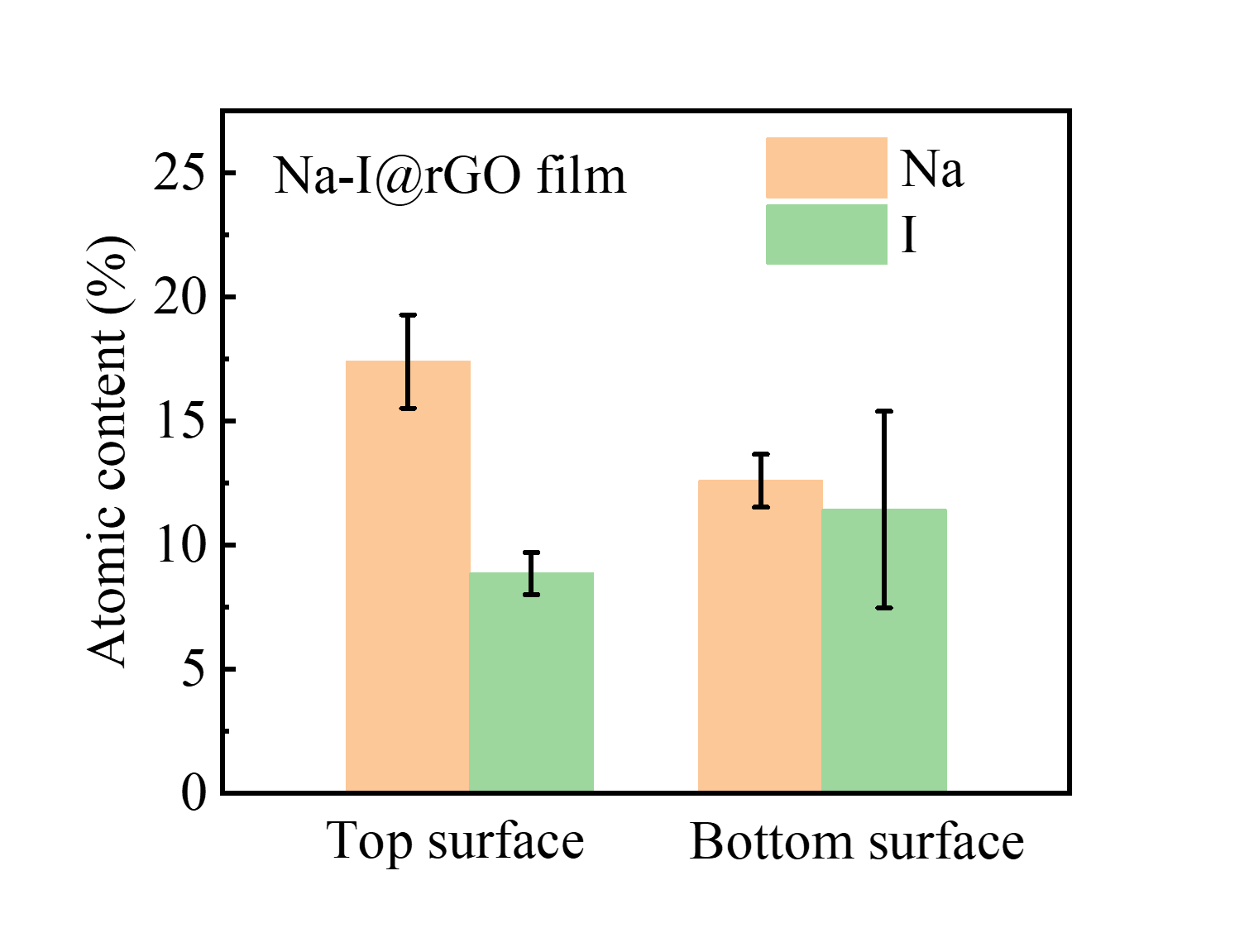


**Figure S1.** Average atomic content of Na and I on the top and bottom surfaces of the Na-I@rGO film determined by SEM-EDS analysis. Selected areas of EDS were randomly chosen from a large sampling region. Error bars indicate the standard deviation from 15 regions.


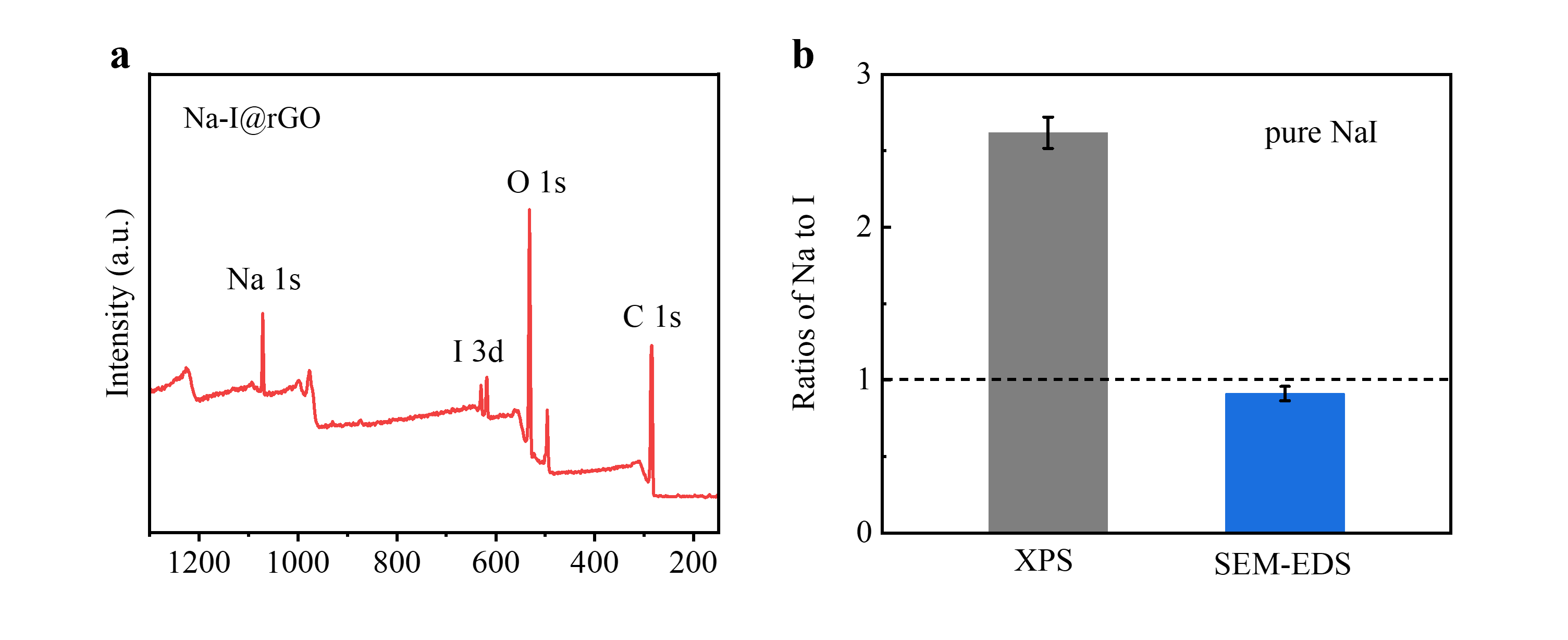


**Figure S2**. **a** XPS spectrum of Na-Cl@rGO film. **b** Atomic ratios of Na to I in pure NaI by XPS and SEM-EDS. Error bars indicate the standard deviation from three regions.


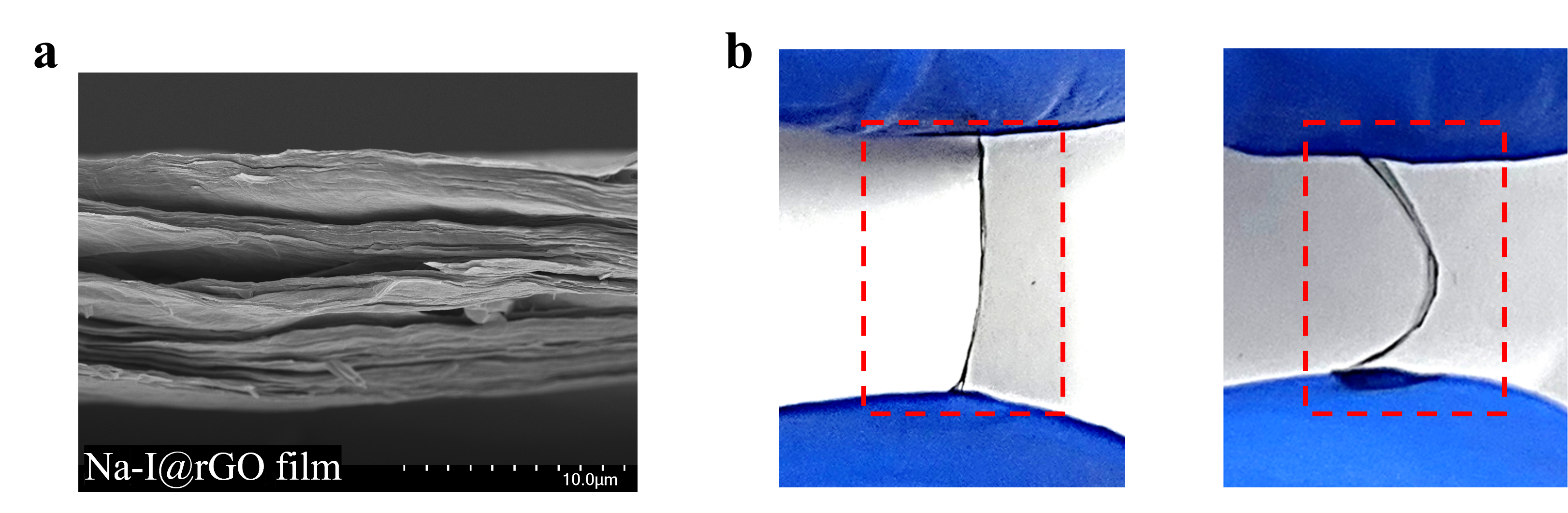


**Figure S3.** **a** Cross-sectional SEM image of the Na-I@rGO film. **b** Photographs of the Na-I@rGO film (1 cm×1 cm) in its pristine and bent states.


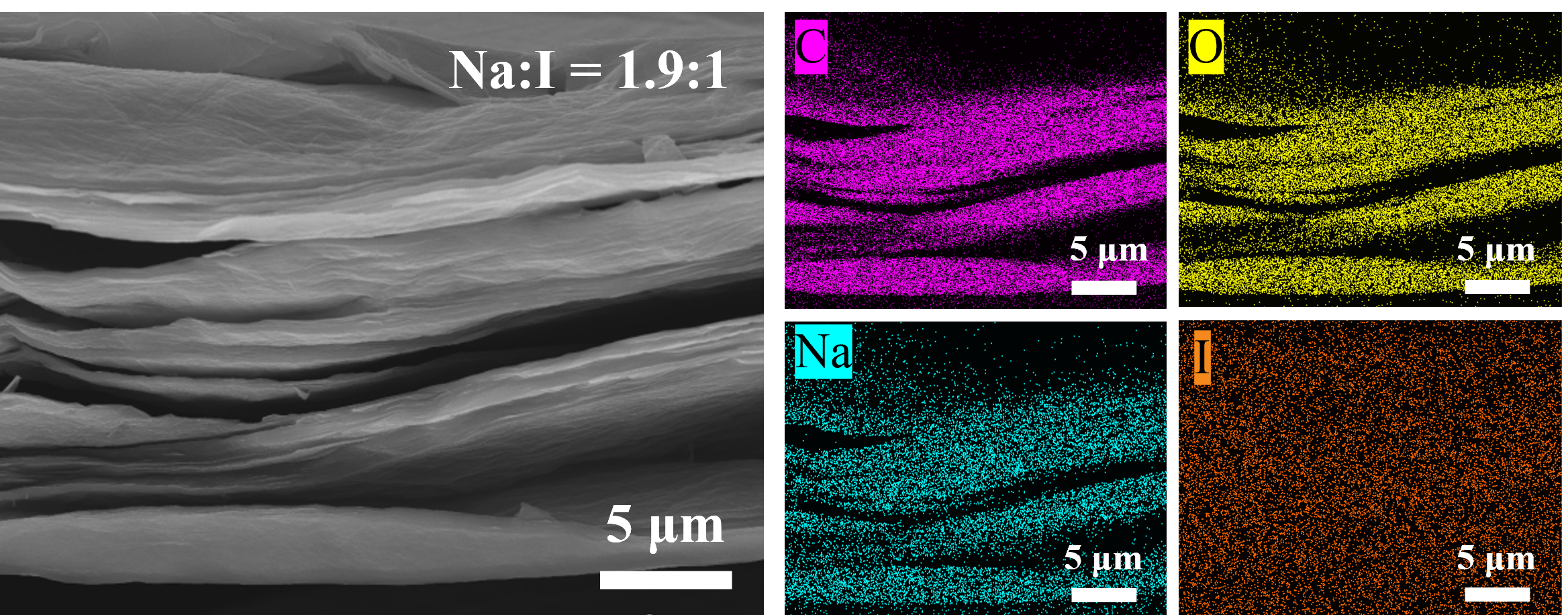


**Figure S4.** SEM image and EDS-Mappings of cross-section of the Na-I@rGO film.


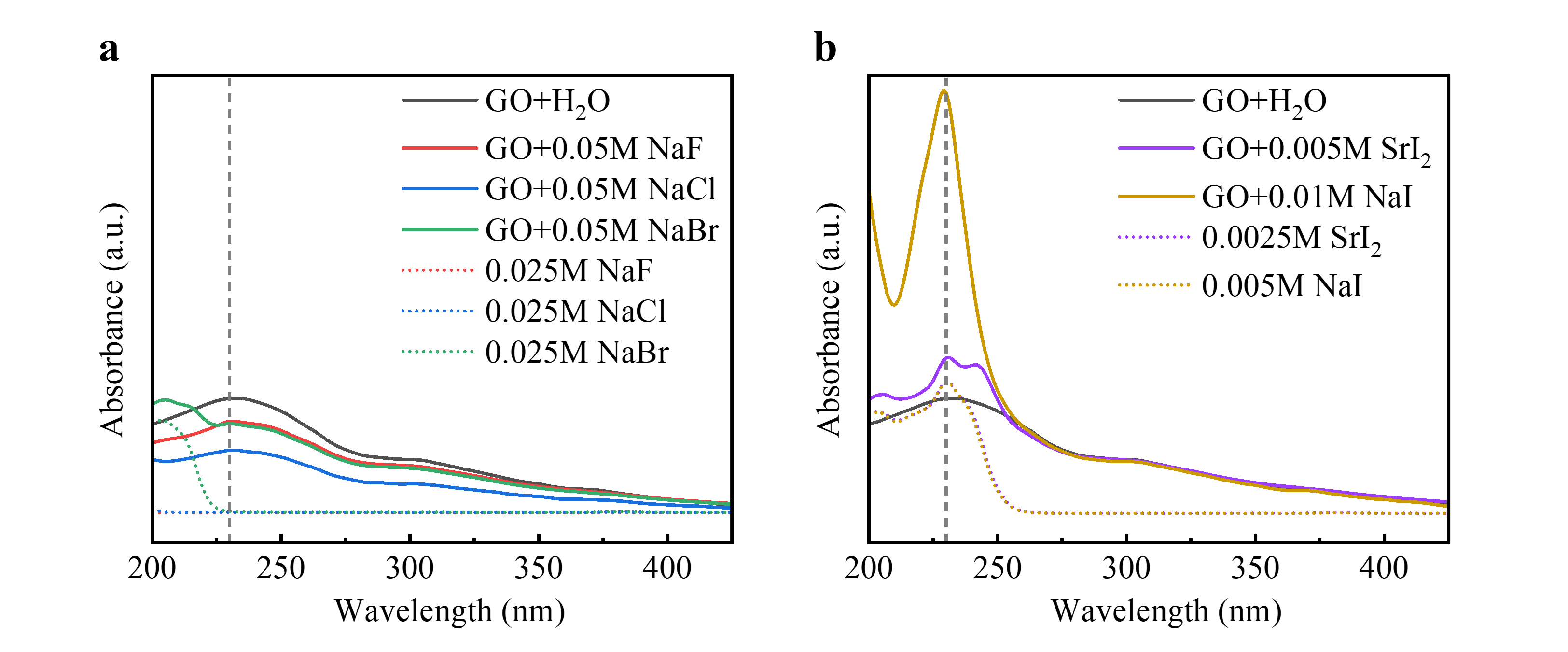


**Figure S5. a** UV spectra of GO suspension (~1 mg/mL) mixed in a 1:1 ratio with NaF, NaCl, and NaBr solutions (red, bule, and green solid line) together with GO suspension in pure water (black solid line), and pure NaF, NaCl, and NaBr solutions (red, bule, and green solid line dashed line). **b** UV spectra of GO suspension (~1 mg/mL) mixed in a 1:1 ratio with NaI and SrI_2_ solutions (yellow and purple solid line) together with GO suspension in pure water (black solid line), and pure NaI and SrI_2_ solution (yellow and purple dashed line).


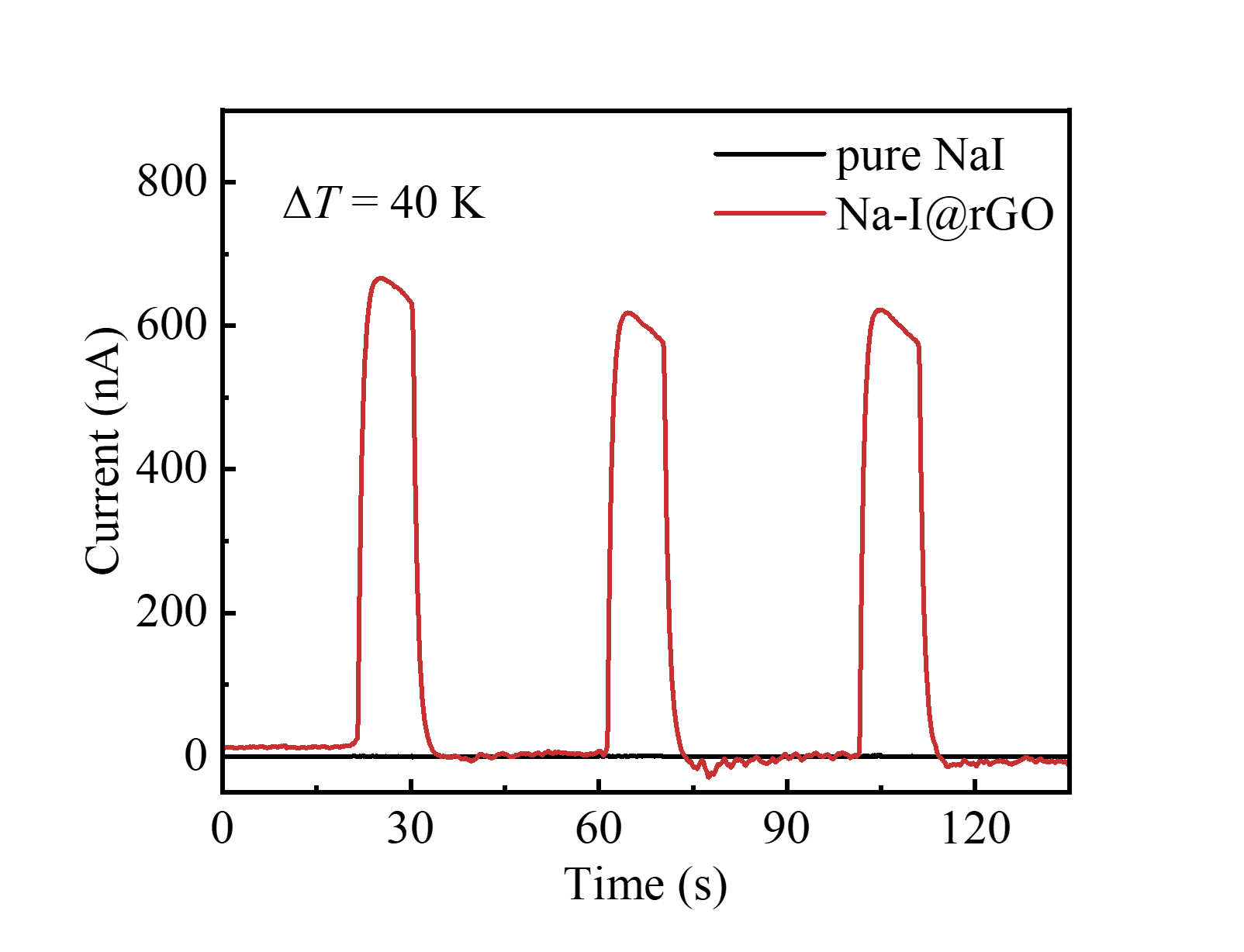


**Figure S6.** Thermoelectric response of the pure NaI and Na-I@rGO films under intermittent heating (Δ*T*=40 K).


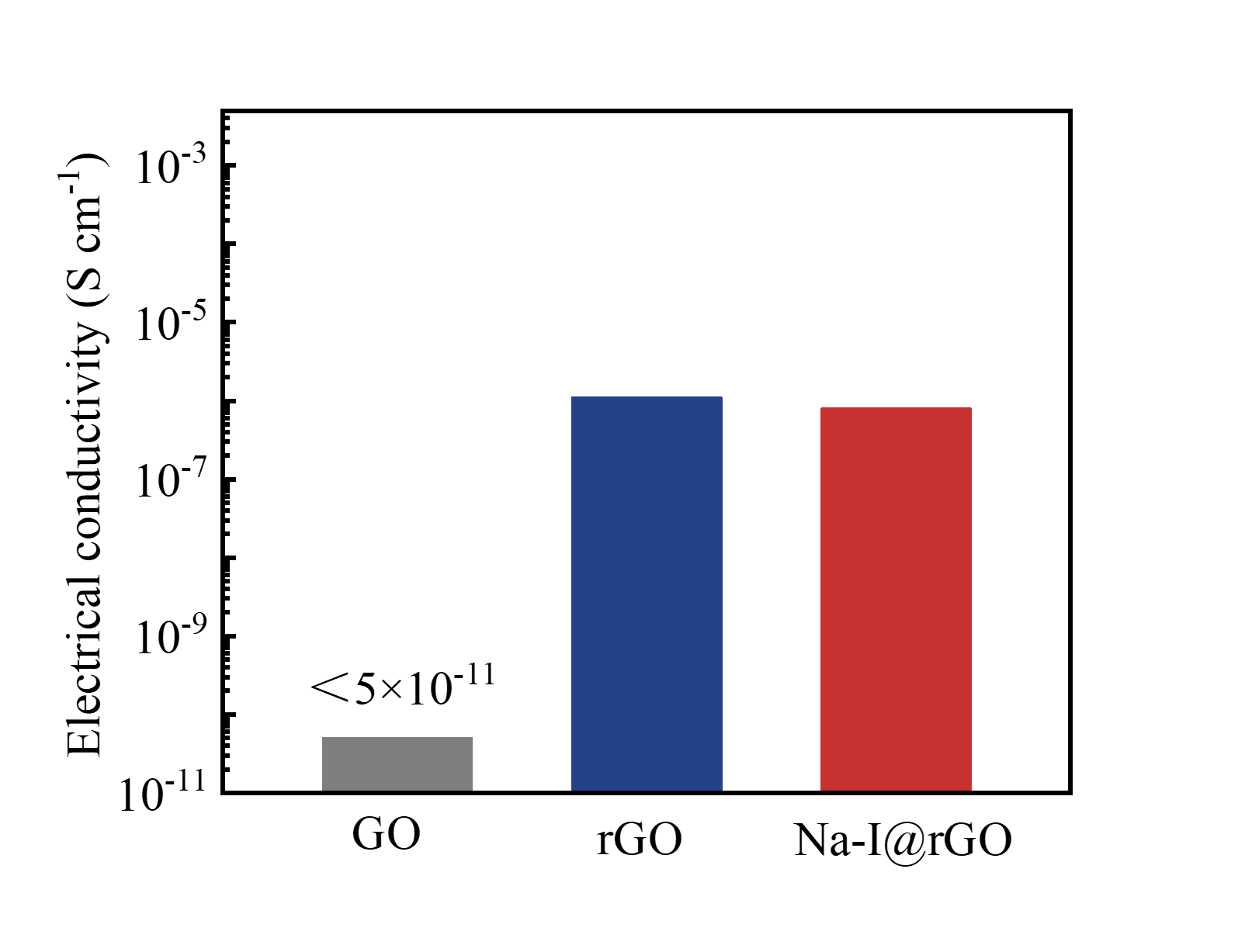


**Figure S7.** Room-temperature electrical conductivity (*σ*) of the GO, rGO, and Na-I@rGO films.


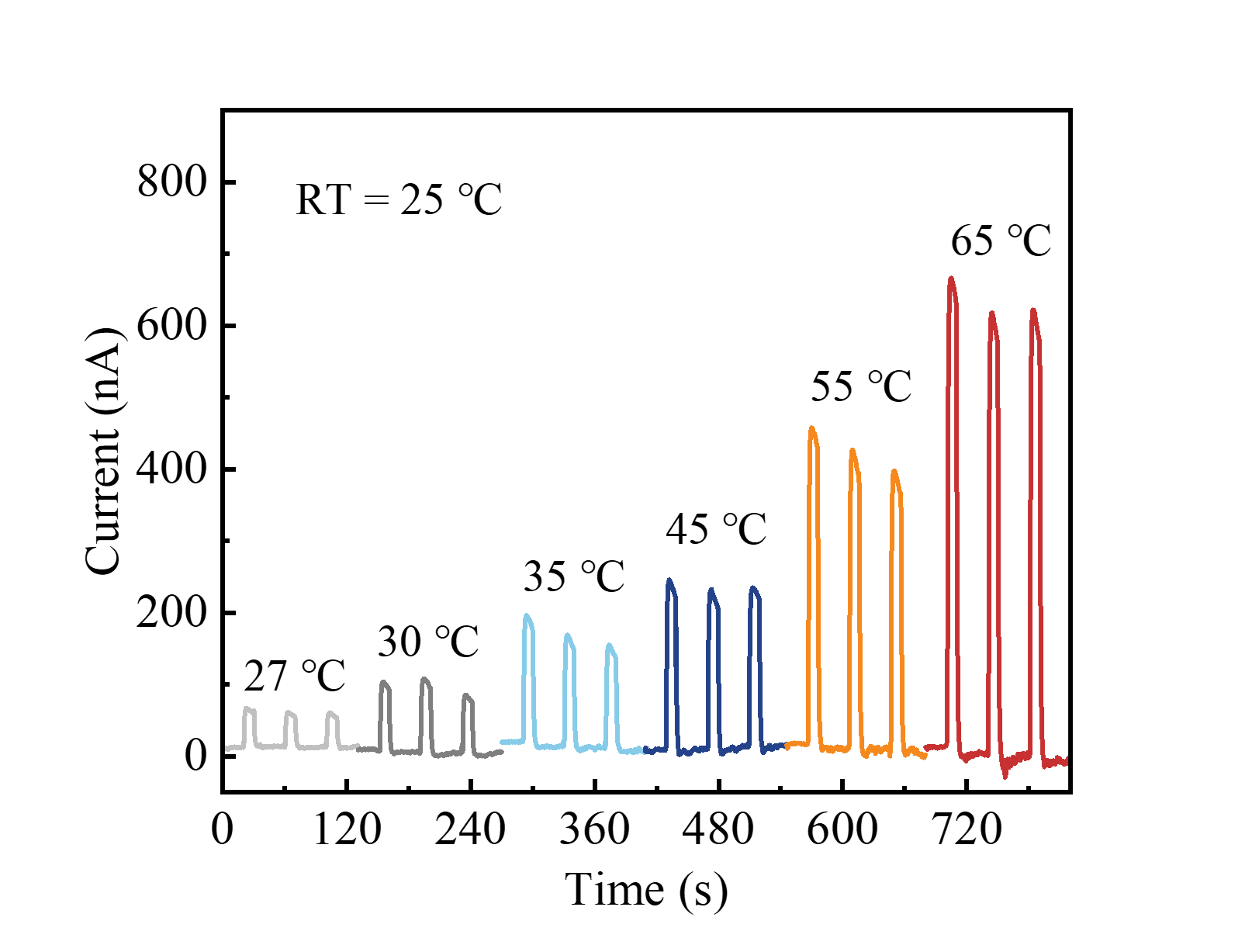


**Figure S8.** Changes in the response current under different temperature of the Na-I@rGO film (compared to room temperature of ~25 ℃).


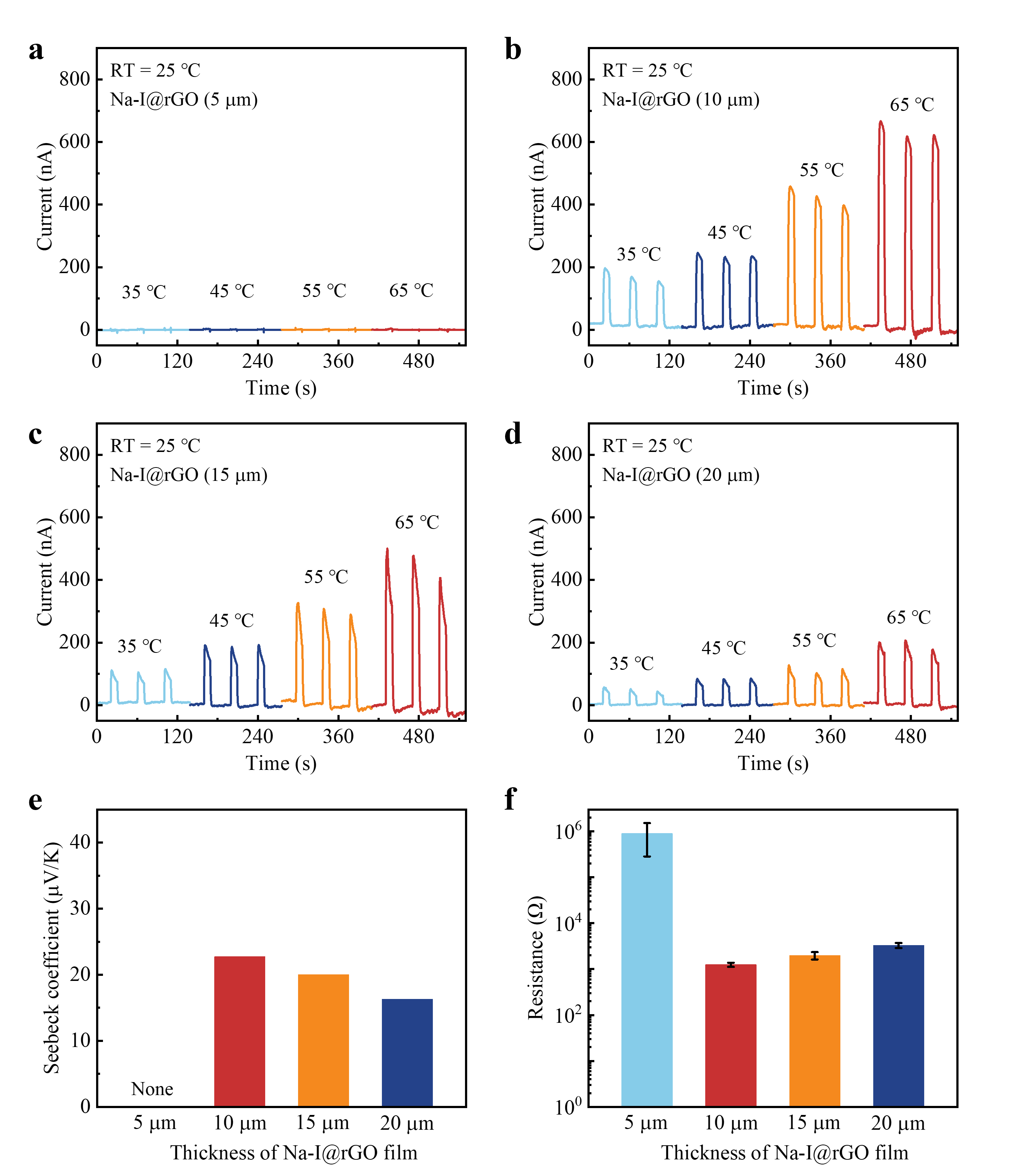


**Figure S9.** Response current of (**a**) 5, (**b**) 10, (**c**) 15, and (**d**) 20 μm Na-I@rGO films under different temperature (compared to room temperature of ~25 ℃). **e** Seebeck coefficient for Na-I@rGO film of different thicknesses by ohmic transformation. **f** Resistance between the up and bottom surfaces for Na-I@rGO films of different thicknesses determined by multimeter. Error bars indicate the standard deviation from five different samples.
